# Reserve and resilience in CKD: concept introduction and baseline results from the Physical REsilience Prediction in Advanced REnal Disease (PREPARED) study

**DOI:** 10.1186/s12882-022-03033-w

**Published:** 2022-12-31

**Authors:** C. Barrett Bowling, Maren K. Olsen, Theodore S. Z. Berkowitz, Battista Smith, Breana Floyd, Nadya Majette, Amy L. Miles, Steven D. Crowley, Virginia Wang, Matthew L. Maciejewski, Heather E. Whitson

**Affiliations:** 1 Durham Veterans Affairs Geriatric Research Education and Clinical Center, Durham Veterans Affairs Health Care System (VAHCS), Durham, NC USA; 2 Center of Innovation to Accelerate Discovery and Practice Transformation, Durham Veterans Affairs Health Care System, Durham, NC USA; 3 Center for the Study of Aging and Human Development (the Aging Center), Duke University, Durham, NC USA; 4 Department of Medicine, Duke University, Durham, NC USA; 5 Department of Biostatistics and Bioinformatics, Duke University, Durham, USA; 6 Department of Population Health Sciences, Duke University, Durham, NC USA

**Keywords:** Chronic kidney disease, Older adults, Resilience, Function, Geriatric nephrology

## Abstract

**Background:**

The purpose of this manuscript is to introduce reserve and resilience as novel concepts in chronic kidney disease (CKD) research and present baseline data from a unique prospective cohort study designed to characterize recovery from functional decline after a health event.

**Methods:**

The Physical REsilience Prediction in Advanced REnal Disease (PREPARED) study recruited a national, prospective cohort of Veterans ≥70 years old with an estimated glomerular filtration rate (eGFR) < 30 ml/min/1.73 m^2^, prior nephrology care, and at high risk for hospitalization. Electronic health record data were paired with telephone surveys. Self-reported measures of reserve included physical, psychological, and cognitive capacity and environmental resources. We calculated counts (frequencies) and medians (25th, 75th percentiles) for baseline measures of reserve. The study’s longitudinal follow-up of physical function every 8 weeks or following an acute care encounter, which will be used to define resilience, is ongoing.

**Results:**

Participants had a median (25th, 75th percentile) age of 76.3 (72.8, 81.4) years and eGFR of 23.4 (18.2, 28.8) ml/min/1.73 m^2^; 23.3% were Black, and 97.4% were male, 91.6% had hypertension, 67.4% had diabetes mellitus, 46.0% had coronary heart disease, and 39.8% had heart failure. Baseline measures of physical, psychological, and cognitive domains showed low reserve on average, but with wide ranges.

**Conclusions:**

Despite similar levels of kidney function, older adults participating in PREPARED had a wide range of measures of reserve in other health domains. Non-renal measures of reserve may be important indicators of capacity of CKD patients to recover after acute care encounters.

## Introduction

Chronic kidney disease (CKD) is common among older adults and associated with functional decline [1–3]. Compared to older adults with an estimated glomerular filtration rate (eGFR) ≥ 60 ml/min/1.73 m^2^, those with an eGFR < 45 ml/min/1.73 m^2^ (Stage ≥ G3b) are more than twice as likely to have worsening activities of daily living (ADL) disability and have a faster decline in life-space mobility, a measure of community mobility and social participation [4, 5]. By the time older adults progress to kidney failure, 50% are dependent in multiple ADLs and nearly 30% require skilled nursing facility care [6].

While prior studies have shown that older adults with advanced CKD are at risk for functional decline, less is known about what contributes to their functional loss. One possibility is that older adults with advanced CKD commonly experience health stressors, including illnesses or injuries that require an acute care encounter such as an emergency department (ED) visit or hospitalization, and these events result in functional impairment [7–9]. One’s capacity to respond before a health stressor and the trajectory of functional recovery afterwards are described by the emerging geriatric concepts of reserve and resilience, respectively [10, 11]. However, reserve and resilience have not yet been studied among older adults with CKD.

The Physical REsilience Prediction in Advanced REnal Disease (PREPARED) study was designed to characterize reserve and resilience in older adults with CKD and identify patient factors associated with greater physical resilience defined as the ability to resist or recover from functional decline after a health event. The purpose of this manuscript is to 1) introduce reserve and resilience in CKD and define key terminology, 2) describe unique data collection methods used in the PREPARED study, and 3) report baseline results on reserve for PREPARED participants.

## Methods

### Physical resilience concepts and terminology

**Fig. 1:**
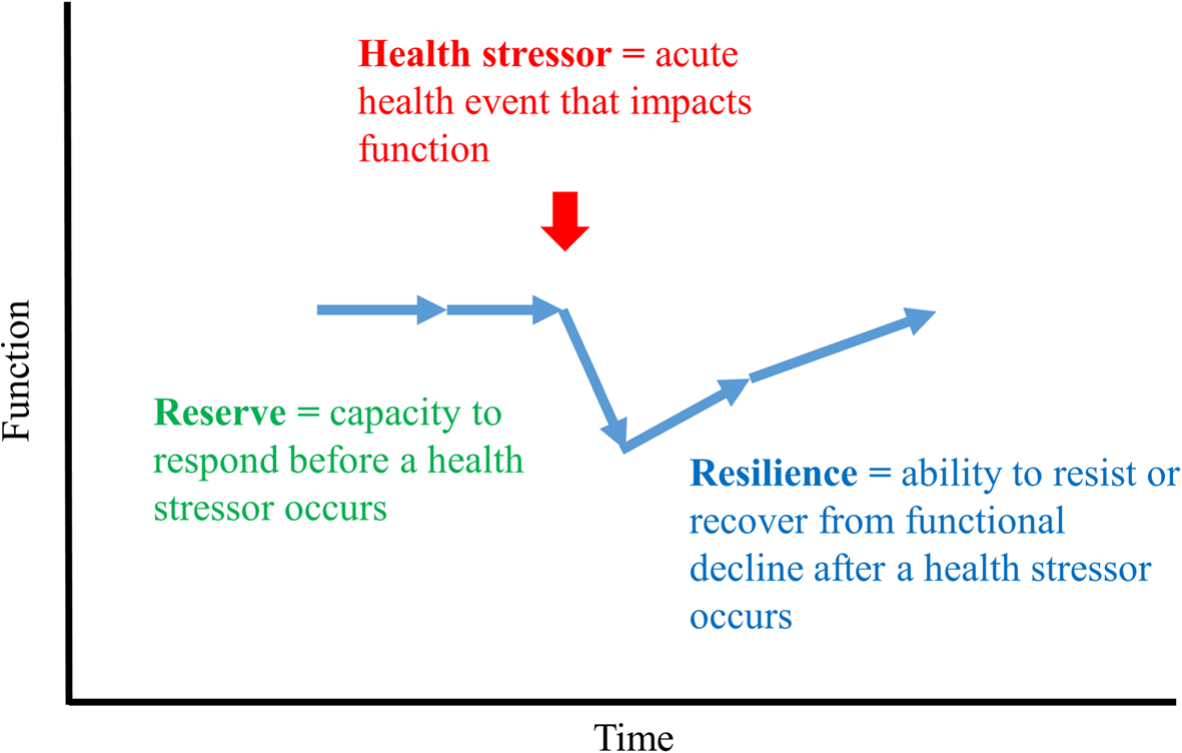
The PREPARED study physical resilience conceptual model. Health stressors include acute health events that impact function, such as an illness or injury. Health stressors often result in increased physical demands. Reserve reflects one’s ability to meet the added demands and can be defined by physical, cognitive, or psychological capacity or available social and environmental resources before a health stressor occurs. Resilience is defined by the trajectory of recovery after a health stressor

In contrast to the well-established construct of psychological resilience, defined as adaptive attitudes and behaviors in response to stressful life events [12, 13], physical resilience is an emerging area of aging research [10]. Physical resilience characterizes one’s physical ability to overcome health stressors and is supported by the recognition that while some older adults “snap back” after an acute health event, many others do not recover physical function quickly or at all [11, 14]. An existing model of physical resilience describes the role of both reserve and resilience in recovering from health events (Fig. 1). In this model, *health stressors* include acute health events that may impact function, such as an illness or injury that results in a hospitalization or ED visit. Health stressors often result in increased physical and cognitive demands. To meet the added demands of a health stressor, older adults may draw on their *reserve* defined as one’s physical, cognitive, or psychological capacity and the social and environmental resources available before a health stressor. The term reserve emphasizes that these abilities are not already in use, but are available when needed. In the model used here, *resilience* is defined by the trajectory of recovery after a health stressor. Resilience is evident when older adults avoid health stressor-related functional loss (i.e., resist) or recover (i.e., snap back) to pre-health stressor levels. Common clinical scenarios in which resilience, or lack of thereof, is evident include when some older adults recover from very severe illness, or some recover better than expected given an apparent limited pre-stressor reserve, or yet others have unexpected functional decline after minor health events. These scenarios suggest that all three factors – health stressors, reserve, and resilience – play a role in functional decline and recovery. These insights informed the PREPARED study of reserve and resilience that includes 1) measures of multiple domains of reserve before a health stressor, 2) prospective identification of health stressors with information on timing and severity, and 3) longitudinal measures of function, both before and after a health stressor, to capture functional decline and recovery [15].

### Study design

**Fig. 2:**
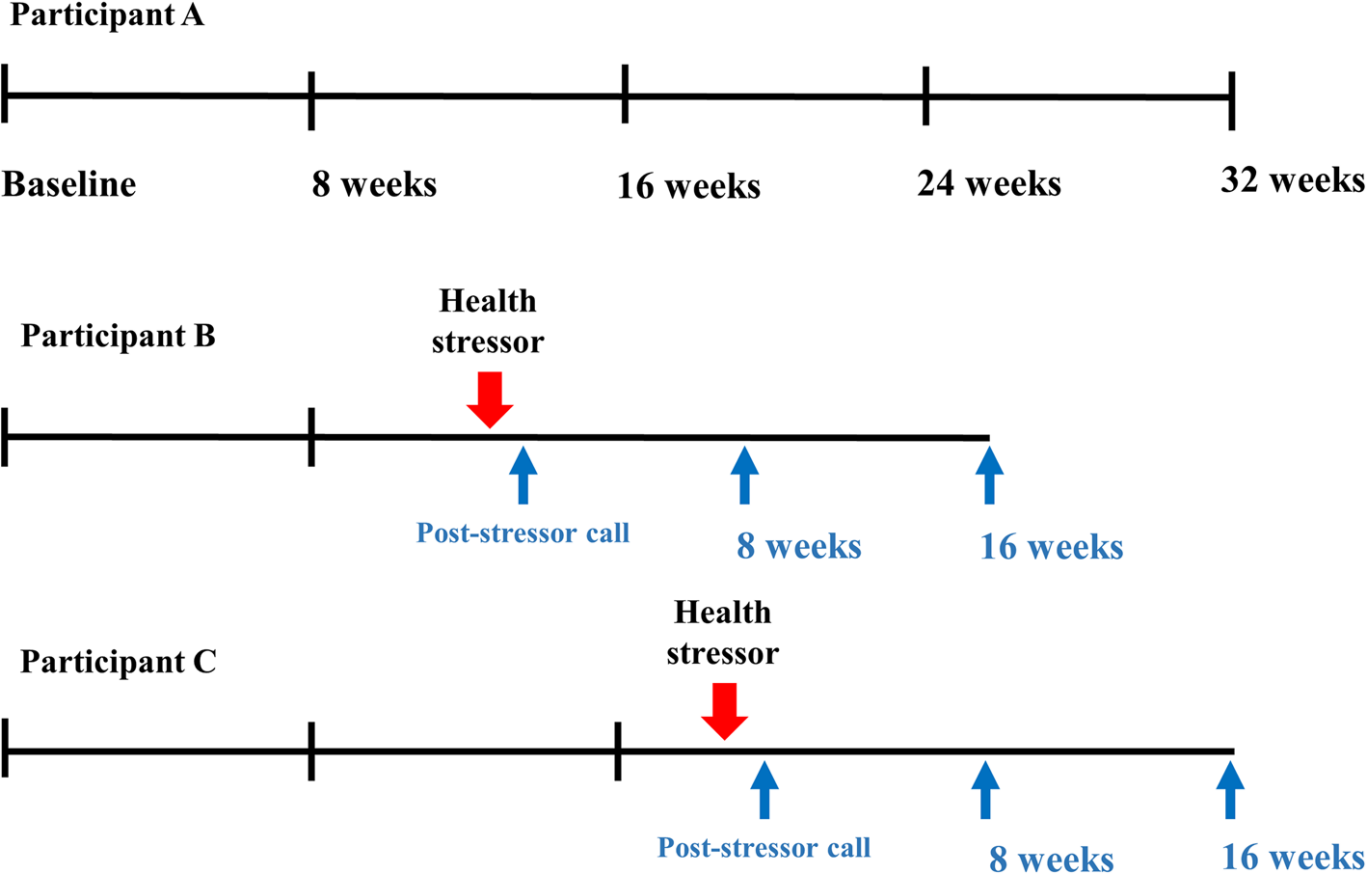
The PREPARED study follow-up schedule. Scheduled telephone surveys occur every 8 weeks for up to 32 weeks. Post-health stressors calls occur within 3–14 days of the health stressor and then every 8 weeks for 2 additional calls. Three example participants with **A**) no health stressor during follow-up, **B**) a health stressor after 8 weeks, and **C**) a health stressor after 16 weeks

PREPARED is a national, prospective cohort study of Veterans with advanced CKD designed to characterize physical resilience. PREPARED uses data from the Department of Veterans Affairs (VA) Health Administrations health system data paired with telephone surveys. The VA Corporate Data Warehouse (CDW) is a national repository of clinical and administrative data that is updated daily and provides detailed information on health care utilization. Primary data were collected through telephone surveys occurring at the time of study enrollment (baseline) to assess reserve and every 8 weeks for up to 32 weeks (8-, 16-, 24-, and 32-week calls) to characterize changes in physical function. On a weekly basis, the study team queried CDW to identify enrolled participants who experienced a health stressor defined as an acute care encounter (ED visit or hospitalization; Fig. 2). Those who had an ED visit without an associated hospitalization were flagged as having a health stressor with the date corresponding to the ED visit. For those hospitalized following the ED visit, we used the hospital discharge date so that the ED visit and hospitalization were considered a single health stressor event. Participants were then contacted within 3 to 14 days of the health stressor and every 8 weeks for 2 additional calls in order to capture their post-stressor functional trajectory.

### Study population

The study cohort comprised older adult Veterans with CKD and at risk for functional decline. Eligible Veterans were identified via CDW who met the following inclusion criteria [1]: age ≥ 70 years old [2], estimated glomerular filtration rate (eGFR) < 30 ml/min/1.73 m^2^ on two occasions at least 90 days apart without an intervening eGFR ≥30 ml/min/1.73 m^2^ [3], prior nephrology consultation or outpatient referral, and [4] high risk for hospitalization based on the VA Care Assessment Need score. We restricted our study population to those with VA laboratory-based eGFR < 30 ml/min/1.73 m^2^ estimated using the CKD-EPI equation (Stage G4 or greater) and prior nephrology consultation in order to identify those most likely to experience CKD-related complications or have had discussions about kidney failure treatment. We restricted our study population to those with a 90-day hospitalization CAN score ≥ 95 in order to identify a group at higher risk for experiencing a qualifying health encounter during follow-up [16, 17].

To focus our study on Veterans with pre-dialysis kidney disease, we excluded those with a history of kidney transplant or those on dialysis. Those with cognitive impairment based on a 6-item telephone screen [18], and those residing in a nursing home or receiving hospice care prior to enrollment were also not eligible for participation. We followed a pre-determined protocol to identify and address hearing limitations in order to avoid excluding those with hearing impairment [19]. We asked all participants to identify and provide permission to contact a proxy if they were no longer able to participate during follow-up.

Enrollment began in October 2019. This study was approved by the Durham VA Institutional Review Board (IRB #2205).

### Measures

#### Measures of pre-stressor reserve.

Measures of baseline physical reserve included life-space mobility, self-reported difficulty with basic and instrumental activities of daily living (ADLs), a symptom burden scale and self-reported falls in the prior year. Life-space mobility was measured using the validated University of Alabama at Birmingham Life Space Assessment which asks how far people go, how often they go there, and how much help they need as they move through five life-space zones [4, 20, 21]. Life-space zones include areas outside of [1] the room where they sleep [2], their home [3], their yard [4], their neighborhood, and [5] their town. Assistance is measured as needing help from a device, such as a cane or walker, or from another person. The composite life-space mobility score incorporates distance (life-space zone), the frequency with which it is attained per week (less than 1 time, 1–3 times, 4–6 times, or daily), and the degree of independence based on the reported use of assistive equipment or help from another person (no assistance > assistive device > personal assistance). Scores range from 0 to 120 with higher scores indicating greater community mobility. Baseline function also included measures of self-reported difficulty with six basic ADLs (bathing, dressing, grooming, toileting, eating, and transferring) and eight IADLs (heavy housework, light housework, shopping, preparing meals, managing money, using the telephone, taking medications, and managing transportation) [22]. The symptom burden score included a count of the following 10 symptoms: shortness of breath, fatigue, dizziness, pain, leg weakness, joint stiffness, nervousness, anhedonia, poor appetite, and constipation [23, 24].

Measures of baseline cognitive and psychological reserve included telephone assessments of cognitive status (modified Telephone Interview for Cognitive Status [TICS-m]) and depressive symptoms (4-item Center for Epidemiologic Studies Depression Scale [CES-D-4]) [25–27].

Measures of personal and environmental resources included education level, marital status, number of household members, social support assessed using the modified Medical Outcomes Survey Social Support scale (mMOS-SS) [28], and a single-item personal economic situation measure which includes five response options characterized as being “in good shape,” “ok,” “barely getting by,” “falling behind,” or “in serious financial trouble.”

#### Other demographic and clinical characteristics.

Demographic factors obtained from CDW included age, gender and geographic region. Self-reported race (American Indian or Alaska Native, Asian, Black or African American, Native Hawaiian or Pacific Islander, White, or other with the option of selecting more than one race) and ethnicity (Hispanic or Latino/a versus not) were obtained by telephone survey. Data on eGFR and body mass index (BMI) were obtained from the most recent CDW lab and vital sign data prior to enrollment. Chronic conditions included hypertension, diabetes mellitus, coronary heart disease, heart failure, and stroke, defined by the presence of inpatient or outpatient ICD-10 diagnosis codes based on recommended algorithms from the Centers for Medicare and Medicaid Services Chronic Conditions Warehouse [29].

### Analysis

We calculated counts (frequencies) and medians (25th, 75th percentiles) for participant characteristics and baseline measures of reserve. To describe overlapping prevalence of low reserve across the many measures we collected (i.e., from cognitive, functional, and psychological domains), we created an UpSet plot for the following seven measures: life-space mobility, ADLs, IADLs, cognition, depressive symptoms, symptom burden, and social support. Results from an UpSet plot are similar to intersecting circles of a Venn diagram, but the UpSet plot is easier to interpret when there are more than three variables included. For each measure, we used the quartile to create a cut-point for the lowest level of reserve (e.g., 75th percentile for ADLs indicates greater difficulty and the 25th percentile for TICS-m indicates worse cognitive function) relative to other PREPARED participants. The UpSet visualization uses dots connected by a line to show what domains are in each intersection. This is displayed at the bottom of the figure. For example, those with only one domain of low reserve will have a single dot. In contrast those with the combination of all 7 domains of low reserve will have seven dots connected by a solid line. The UpSet visualization also displays the number of participants within each intersection (vertical bars labeled “Number of Participants”).

## Results

Of the 417 participants in PREPARED, there was a median age (25th, 75th percentile) of 76.3 (72.8, 81.4) years, 23.3% were Black, and 97.4% were male (Table 1). Participants had a median eGFR of 23.4 ml/min/1.73 m^2^ (18.2, 28.8) and 91.6% had hypertension, 67.4% had diabetes mellitus, 46.0% had coronary heart disease, and 39.8% had heart failure.

**Table 1:** Baseline characteristics of PREPARED participants (*n* = 417)

Characteristic	Median (25th, 75th percentile) or N (%)
Age	76.3 (72.8, 81.4)
Race	
American Indian or Alaska Native	3 (0.7%)
Asian	2 (0.5%)
Native Hawaiian or Pacific Islander	3 (0.7%)
Black or African American	97 (23.3%)
White	275 (65.9%)
More than one race	19 (4.6%)
Not reported	18 (4.3%)
Male	406 (97.4%)
Geographic region	
Northeast	59 (14.1%)
South	197 (47.2%)
Midwest	82 (19.7%)
West	79 (18.9%)
eGFR, ml/min/1.73 m^2^	
CKD-EPI (original)	23.4 (18.2, 28.8)
CKD-EPI (2021)	24.6 (19.1, 30.1)
BMI, kg/m^2^	30.5 (26.8, 34.6)
Hypertension	382 (91.6%)
Diabetes mellitus	281 (67.4%)
Coronary heart disease	192 (46.0%)
Heart failure	166 (39.8%)
Stroke	26 (6.2%)

*PREPARED *Physical REsilience Prediction in Advanced REnal Disease, *eGFR *Estimated glomerular filtration rate, *BMI *Body mass index, *CKD-EPI *Chronic Kidney Disease Epidemiology Collaboration

Baseline life-space mobility showed a wide range: median 52.0 (35.0, 72.0) (Table 2). These findings are consistent with participants in the lowest quartile of life-space mobility being restricted to areas within their yard and participants in the highest quartile going to areas beyond their town. Median (25th, 75th percentile) scores for difficulty with ADLs and IADLs were 1.0 (0.0, 2.0) and 3.0 (1.0, 6.2), respectively, indicating a range of functional levels among PREPARED participants at baseline. Participants frequently reported a fall in the prior year (50.4%) and individual symptoms ranged in prevalence from fair or poor appetite (28.1%) to feeling fatigued or tired (78.4%).

**Table 2:** Baseline measures of reserve for PREPARED participants

Physical reserve	Median (25th, 75th percentile) or N (%)
ADL difficulty, range 0 to 18	1.0 (0.0, 2.0)
IADL difficulty, range 0 to 24	3.0 (1.0, 6.2)
Life-space mobility	52.0 (35.0, 72.0)
Number of chronic conditions (Multimorbidity)	
0	14 (3.4%)
1	68 (16.3%)
2	121 (29.0%)
3	128 (30.7%)
4–5	86 (20.6%)
Falls	210 (50.4%)
Symptoms	
Nerves/nervousness (≥sometimes)	168 (40.3%)
Little interest or pleasure (≥sometimes)	186 (44.6%)
Appetite (fair or poor)	117 (28.1%)
Pain (≥4–6/week)	270 (64.7%)
Shortness of breath	210 (50.4%)
Tired/fatigued	327 (78.4%)
Balance/dizziness	229 (54.9%)
Leg weakness	256 (61.4%)
Constipation	143 (34.3%)
Stiffness	253 (60.7%)
Symptom burden score	5.0 (3.0, 7.0)
**Cognitive/psychological reserve**	
Cognition score (TICS-m), range 0 to 50	32.0 (28.0, 35.0)
Depression score (CESD-4), range 0 to 12	1.0 (0.0, 3.0)
**Personal and environmental resources**	
Education	
Grade school/junior high	10 (2.4%)
Some high school	29 (7.0%)
High school/equivalent	100 (24.0%)
Trade/technical/vocational school	36 (8.6%)
Some college	110 (26.4%)
Associate’s degree	45 (10.8%)
Bachelor’s degree	54 (12.9%)
Post graduate work/graduate degree	33 (7.9%)
Marital status	
Married	217 (52.0%)
Divorced/separated	85 (20.4%)
Widowed	67 (16.1%)
Single, never married	48 (11.5%)
Lives alone (number household members = 1)	133 (31.9%)
Social support (mMOS-SS), range 0 to 100	87.5 (62.5, 100.0)
Financial stress	
In good shape	91 (21.9%)
Ok	225 (54.0%)
Barely getting by	85 (20.4%)
Falling behind	9 (2.2%)
Serious financial trouble	7 (1.7%)

*PREPARED *Physical REsilience Prediction in Advanced REnal Disease, *ADL *Activities of daily living, *IADL *Instrumental activities of daily living, *TICS-m *Modified Telephone Interview for Cognitive Status, *CESD-4 *Item Center for Epidemiologic Studies Depression scale, *mMOS-SS *Modified Medical Outcomes Survey Social Support scale

Measures of cognitive and psychological reserve showed a range of cognitive scores and depressive symptoms. Ranges of response to measures of personal and environmental resources across education, marital status, social support and financial stress were also found with 33.3% reporting high school education or less, 48.0% being divorced/separated, widowed, or never married, 31.9% living alone, and 24.2% reporting barely getting by or worse financially.

**Fig. 3:**
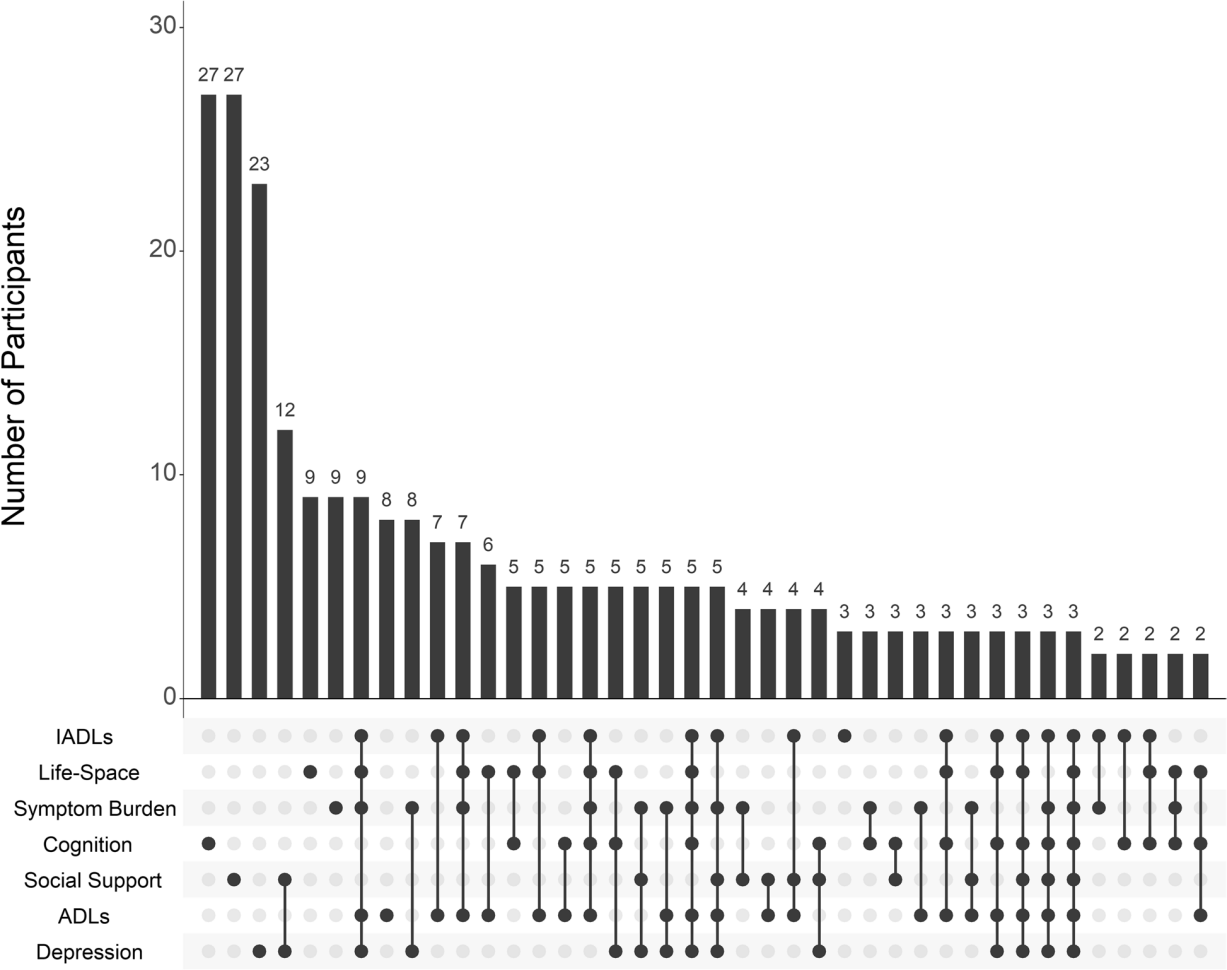
UpSet visualization showing overlapping domains of low reserve for PREPARED participants. Low reserve in social support, life-space mobility, and cognition defined as lowest quartile of scores. Low reserve in ADLs, depressive symptoms, IADLs and symptom burden defined as the highest quartile of scores. Figure truncated at 40 intersections. Groups of overlapping domains are shown as dots (single domain such as cognition) and dots connected by lines (more than 1 domain such as depression + social support). The number of participants in each group is shown by vertical bars labeled “Number of Participants” (e.g., 27 participants had only low reserve in cognition)

Patterns of single and overlapping domains of low reserve are shown in Fig. 3. Overall, there were 93 unique patterns of overlapping domains of low reserve. The most common patterns were low cognition (27 participants with TICS-m ≤ 25th percentile) and low social support (27 participants with mMOS-SS ≤ 25th percentile), both with no other domains of low reserve. Nine participants had the combination of low reserve in IADLs, life-space mobility, symptom burden, ADLs, and depressive symptoms (7th bar from the left) and three participants had the combination of all seven domains of low reserve (35th bar from the left).

## Discussion

Older age has been characterized as a time of progressive loss in physical function; however, functional impairment can be a dynamic process with episodes of decline following a health event and subsequent recovery. This process of functional decline and recovery in the face of health stressors has been operationalized in recent aging research through the concept of physical resilience. As older adults with advanced CKD often experience health stressors, understanding reserve and resilience in this population may be important for predicting which patients are at highest risk of sustained functional decline. The PREPARED study was designed to characterize physical resilience among older adults with CKD using structured longitudinal surveys paired with clinical and health care utilization data from the largest integrated health care system in the US. Longitudinal surveys are underway and will be used to collect data on physical function before and after a health stressor (if one occurred) and measures of physical, psychological and cognitive reserve, as well as personal and environmental factors that are not fully captured in electronic health record data.

Descriptive analyses of baseline characteristics and measures of reserve showed that even in this group of older Veterans with the same stage of CKD (i.e., KDIGO G4, eGFR interquartile range 11 ml/min/1.73 m^2^), there was variability across measures of reserve. This variability was seen for measures of function (e.g., life-space mobility, ADLs), number of chronic conditions, falls, symptom burden, cognition, depression, psychological resilience, education, marital status, social support, and financial stress. An approach that relies solely on kidney disease biomarkers to risk stratify and guide clinical management, may not adequately recognize the unique sets of strengths and challenges that influence one’s ability to respond to acute health events.

Another finding with potential clinical implications was the many different combinations of measures of low reserve displayed in Fig. 3. Each of the different combinations is shown by a separate vertical bar with the dots and lines below indicating which measures of reserve were included in that combination. As shown in the figure, some participants had low reserve in a single measure (e.g., 27 participants with low cognition alone) and others experienced low reserve in multiple measures (e.g., 9 participants with low reserve in physical function, depressive symptoms, and symptom burden). When providing routine clinical care for this population, nephrologists and primary care physicians may need to tailor care to the individual needs of patients. For example, challenges to CKD self-management (i.e., BP monitoring, low potassium diet) and strategies to address challenges (i.e., written instructions, including a caregiver) may be very different for a patient experience only cognitive impairment (first vertical bar in Fig. 3) versus a patient with high symptom burden and depression (9th vertical bar in Fig. 3) versus a patient with functional impairment and low social support (23rd vertical bar in Fig. 3). Additionally, future research studies designed to characterize reserve among older adults with advanced CKD may require evaluation across multiple domains.

The PREPARED study has several innovative aspects including the focus on reserve and resilience and pairing EHR and survey data to characterize this complex concept. Additionally, using near-real time identification of health stressors to then trigger additional survey calls is a unique feature of the prospective study design. There are potential limitations that must also be considered. Our study population is comprised primarily of male Veterans. In order to enroll a national sample, it was not feasible to include measures of physical performance such as gait speed. While we restricted our sample to those receiving care in the VA health system through our inclusion of those with past nephrology care and lab values necessary to calculate eGFR, some Veterans seek care for acute health events outside of the VA system and dual use (VA and Medicare) is reportedly common among older Veterans. We did, however, collect self-reported data on health stressors at non-VA hospitals as well as life stressors (e.g., death of a spouse) but recall bias may result in under-estimates. A major limitation of the current, cross-sectional analysis was the lack of a younger comparison group with normal kidney function. Therefore, we could not assess for independent associations of age and reduced kidney function with the domains of reserve. However, prior research that provided the rationale for the current study, has shown consistent associations between reduced kidney function and geriatric syndromes such as low life-space mobility, impairment in activities of daily living, and cognitive impairment [4, 5, 7, 8]. Lastly, data collection occurred during the COVID-19 pandemic. We have previously shown that social distancing may have had unintended consequences in the PREPARED study population and it is possible that older adults delayed care for health stressors during this time [30]. To address this, we extended our enrollment window to ensure as large a sample size as possible.

The PREPARED study used an innovative design to characterize reserve and resilience among older adults with advanced CKD that included longitudinal surveys of function initiated after health stressors. Baseline findings reported here suggest that, despite similar levels of kidney function, older adults participating in PREPARED had a wide range of measures of physical, psychological, and cognitive reserve and personal and environmental resources. Measurement of multiple domains of reserve could provide contextual information on the unique strengths and challenges of individuals within this population. On completion of follow-up data collection, the PREPARED study team will be positioned to describe functional trajectories after a health stressor to characterize resilience. Our future work to determine which pre-stressor reserve factors are most associated with resilience after a stressor may lead to interventions to optimize reserve and resilience among older adults with kidney disease.

## Data Availability

Data sets can be obtained from the corresponding author upon reasonable request and following VA requirements for a written agreement prohibiting the recipient from identifying or re-identifying any individual whose data are included in the dataset.
